# Local Application of Minimally Manipulated Autologous Stromal Vascular Fraction (SVF) Reduces Inflammation and Improves Bilio-Biliary Anastomosis Integrity

**DOI:** 10.3390/ijms26010222

**Published:** 2024-12-30

**Authors:** Ilya Klabukov, Garnik Shatveryan, Nikolay Bagmet, Olga Aleshina, Elena Ivanova, Victoria Savina, Ilmira Gilmutdinova, Dmitry Atiakshin, Michael Ignatyuk, Denis Baranovskii, Peter Shegay, Andrey Kaprin, Ilya Eremin, Nikita Chardarov

**Affiliations:** 1 Department of Regenerative Medicine, National Medical Research Radiological Centre of the Ministry of Health of the Russian Federation, Koroleva st. 4, 249036 Obninsk, Russia; 2 Petrovsky National Research Centre of Surgery, Abrikosovsky per. 2, 119991 Moscow, Russia; 3 National Medical Research Center for Rehabilitation and Balneology of the Ministry of Health of the Russian Federation, Novyy Arbat Str. 2, 121099 Moscow, Russia; 4 Scientific and Educational Resource Center for Innovative Technologies of Immunophenotyping, Digital Spatial Profiling and Ultrastructural Analysis, Patrice Lumumba Peoples Friendship University of Russia (RUDN University), 117198 Moscow, Russia; 5 Department of Biomedicine, University Hospital Basel, Basel University, 4001 Basel, Switzerland; 6 Research and Educational Resource Center for Cellular Technologies, Patrice Lumumba Peoples Friendship University of Russia (RUDN University), 117198 Moscow, Russia

**Keywords:** bile duct, bilio-biliary anastomosis, cholecystectomy, regenerative medicine, stromal vascular fraction, surgery, tissue engineering

## Abstract

Bilio-biliary anastomosis (BBA) is a critical surgical procedure that is performed with the objective of restoring bile duct continuity. This procedure is often required in cases where there has been an injury to the extrahepatic bile ducts or during liver transplantation. Despite advances in surgical techniques, the healing of BBA remains a significant challenge, with complications such as stricture formation and leakage affecting patient outcomes. The stromal vascular fraction (SVF), a heterogeneous cell population derived from adipose tissue, has demonstrated promise in regenerative medicine due to its rich content of stem cells, endothelial progenitor cells, and growth factors. The objective of this study was to evaluate the potential of locally administered autologous SVF to enhance the healing of BBAs. Bilio-biliary anastomosis was performed on a swine model (female Landrace pigs). Six swine were divided into two groups: the treatment group (*n* = 3) received a local application of autologous SVF around the anastomosis site immediately following BBA formation, while the control group (*n* = 3) received saline. The primary outcomes were assessed over an eight-week period post-surgery, and included anastomosis healing, stricture formation, and bile leakage. Histological analysis was performed to evaluate fibrosis, angiogenesis, and inflammation. Immunohistochemistry was conducted to assess healing-related markers (CD34, α-SMA) and the immunological microenvironment (CD3, CD10, tryptase). The SVF-treated group exhibited significantly enhanced healing of the BBA. Histological examination revealed increased angiogenesis and reduced fibrosis in the SVF group. Immunohistochemical staining demonstrated higher vascular density in the anastomosed area of the SVF-treated group (390 vs. 210 vessels per 1 mm^2^, *p* = 0.0027), as well as a decrease in wall thickness (1.9 vs. 1.0 mm, *p* = 0.0014). There were no statistically significant differences in mast cell presence (*p* = 0.40). Immunohistochemical staining confirmed the overexpression of markers associated with tissue repair. Local injections of autologous SVF at the site of BBA have been demonstrated to significantly enhance healing and promote tissue regeneration. These findings suggest that SVF could be a valuable adjunctive therapy in BBA surgery, potentially improving surgical outcomes. However, further investigation is needed to explore the clinical applicability and long-term benefits of this novel approach in clinical practice as a minimally manipulated cell application.

## 1. Introduction

Surgical treatment of various benign and malignant diseases in the hepato-pancreato-duodenal area often involves the creation of different types of anastomoses, especially biliodigestive and bilio-biliary anastomosis. Bilio-biliary and biliodigestive anastomosis strictures are one of the most common complications after liver transplantation, with an incidence ranging from 4 to 25% [1,2,3]. The management of this complication, whether endoscopic or surgical, is time-consuming, often requires repeated interventions, and significantly impairs patients’ quality of life. Nevertheless, the field of tissue-engineered biliary substitutes remains in its infancy [4,5,6]. There has been a notable increase in the use of supportive surgical techniques with the aim of improving outcomes and avoiding post-surgical complications.

The history of biliary injury repair has been marked by significant advances aimed at achieving uncomplicated healing [7,8,9]. Despite the use of artificial materials, autologous and allogeneic tissues, cell technologies, and tissue-engineered constructs, an ideal artificial bile duct substitute has yet to be identified [3,10,11]. Consequently, research focused on non-complicated bile duct healing remains of paramount interest. Recent studies in regenerative medicine have increasingly focused on understanding the immunological responses associated with transplantation and various therapeutic interventions [12,13].

Ischemia in the area of bilio-biliary anastomosis (BBA) has been proposed as a potential cause of stricture formation. This complication can result in the development of life-threatening conditions, including intra-abdominal and intrahepatic abscesses, cholangitis, and sepsis, which may ultimately lead to patient mortality [14,15]. Consequently, the development of prophylactic methods for the insufficiency and strictures of these anastomoses is a highly relevant and urgent research topic.

The repair of bile duct injury is a complex process that involves several cell types, primarily cholangiocytes and resident immune cells [16]. In addition to M1/M2 macrophages, mast cells play an important role in the immune system, both by promoting and resolving inflammation [17]. Mast cells secrete pro-inflammatory cytokines, such as TNF-α and IL-6, to recruit neutrophils and macrophages to the site of injury. Furthermore, they can facilitate cholangiocyte proliferation and survival, thereby promoting bile duct regeneration [16,18,19].

In recent years, the potential use of mesenchymal stem cells (MSCs), particularly adipose-derived mesenchymal stem cells (AD-MSCs), in the treatment of various diseases and injuries has been actively explored [20,21]. The attractiveness of this particular type of MSC for clinical use is attributed to their relative ease of procurement, their abundance (the concentration of MSCs in adipose tissue is 100 times greater than in bone marrow), their higher proliferative activity [22], and their ability to be used in the treatment of various diseases and injuries [23,24]. The process of obtaining MSCs involves the isolation of the stromal vascular fraction (SVF) followed by the cultivation of nucleated cells in a nutrient medium.

The adipose tissue-derived SVF is a complex of cellular populations, including adipose tissue stromal cells (MSCs), vascular endothelial, smooth muscle cells and their progenitors, pericytes, fibroblasts, tissue macrophages, and blood cells (erythrocytes and leukocytes). Currently, the SVF is performed in plastic and reconstructive surgery for accelerating healing processes and reducing the risks of rejection due to ischemic complications. The SVF’s paracrine properties lead to stimulation of the neovascularization and suppress inflammation [25], therefore finding the applications in traumatology and orthopedics [26]. The use of SVF as minimally manipulated cells reduces the regulatory burden in the clinic and significantly decreases the risk of adverse events or side effects development [27,28]. It has also demonstrated the ability to perform intraoperative separation as well as in situ tissue engineering by processing and application of minimally manipulated cells in the Operating Room [29,30]. Previous studies have primarily focused on the broader application of MSCs in liver and biliary repair, with promising results in enhancing tissue regeneration and reducing inflammation. MSCs have been shown to improve outcomes in hepatic fibrosis in animal models, suggesting their potential utility in related surgical applications. Investigation into the application of autologous stem cell sheets to improve biliary anastomosis was performed [31], but previous studies did not assess the presence of immune cells and their responses [32,33], which are important for understanding the underlying mechanisms of uncomplicated anastomotic healing. Consequently, the effects of local applications of AD-MSCs on the healing of bilio-biliary anastomosis remain inadequately investigated, with a paucity of preclinical studies.

The aim of this study was to evaluate the local SVF application on healing and improving the integrity of the bilio-biliary anastomosis in a porcine model, as swine serves as a relevant model for the human biliodigestive system in bile duct size.

## 2. Results

### 2.1. SVF Characterization

The average number of isolated nucleated cells was 0.99 ± 0.10 × 10^6^ with a viability of 92.6 ± 2.3% (Table 1).

The obtained cell culture was characterized by a high proliferative potential, with an average doubling time of 23.3 ± 1.5 h.

### 2.2. Follow-Up Assessment

**Figure 1:**
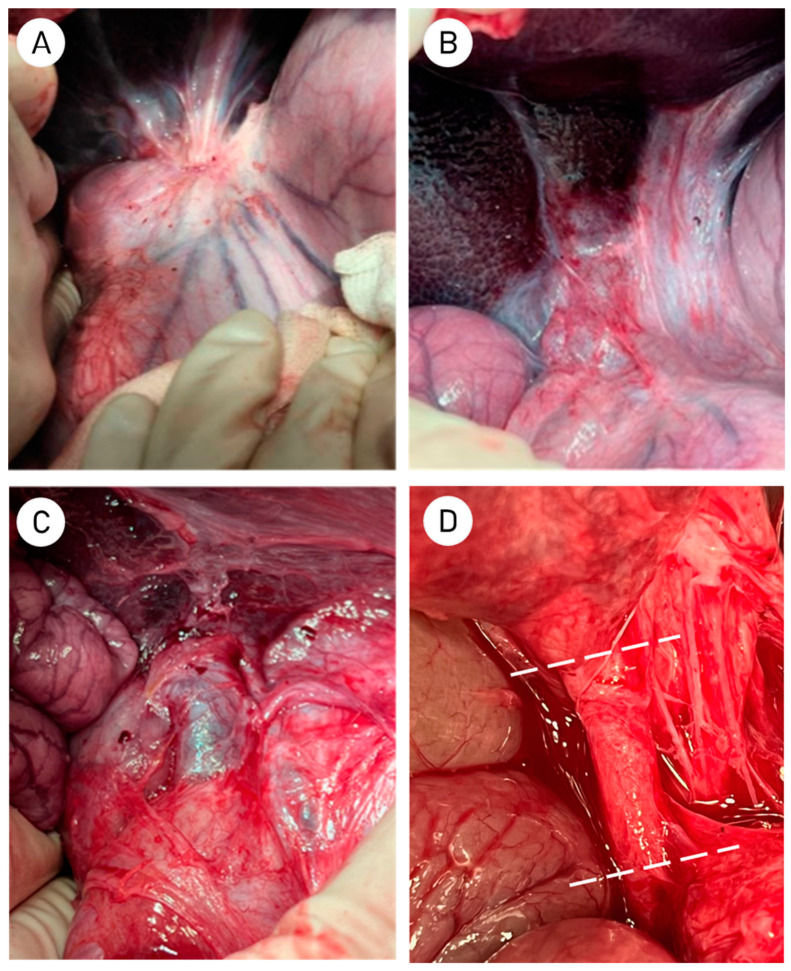
(**A**) View of the subhepatic space and the area of the hepato-duodenal ligament in control group; (**B**) Subhepatic space in SVF-treated group; (**C**) Bile duct in control group; (**D**) Close-up of the anastomosis area in the SVF-treated group with dotted lines indicating the level of bile duct transection.

The surgical procedure lasted for a total of 130 ± 8.9 min. No postoperative complications were observed. Bile admixtures were not present in the drainage output. Drains were removed 6 ± 1.5 days after surgery. After 64.5 ± 3 days after initial surgery, the second stage was carried out. Exposure and examination of the subhepatic space did not reveal localized bile collections or abscesses. In the area of the hepatoduodenal ligament, a moderate fibrous adhesion process was observed, which did not differ between the two groups (Figure 1A,B).

Subsequently, the hepato-duodenal ligament was dissected, and the common hepatic and common bile ducts were isolated in a longitudinal manner. The area of the ductal anastomosis was then examined and photographed (Figure 1A–D). No significant differences were observed in the condition of the surrounding tissues (Figure 1B–D). To prepare the bile duct for morphological studies, the bile duct was resected at a distance of 1–1.5 cm proximal and 2–3 cm distal to the anastomosis (Figure 1D).

### 2.3. Macroscopic Observation

The diameter of the bile ducts in the SVF-treated and control groups was 5.5 ± 1.0 mm and 8.0 ± 2.5 mm, respectively, but there were no significant differences. The duct wall thickness in the SVF-treated group was up to 1 mm, while in the control group, it was 1 mm, with areas of thickening up to 2 mm. The internal lining of the ducts in both groups was smooth and of a whitish-pink color.

### 2.4. Histological Staining Studies

The histologic examination of common hepatic duct anastomosis specimens from the control group, stained with hematoxylin and eosin, Van Gieson’s picrofuchsin, and Mallory’s picric acid, revealed a notable increase in fibrotic alterations within the adjacent connective tissue. The collagen fibers were observed to be dense and multidirectional, accompanied by a limited number of proliferating fibroblasts within the thickness (IHC with antibodies to α-SMA). In the SVF-treated group, fibrotic changes in the anastomotic area were mild, with collagen fibers appearing loose and thin, exhibiting focal myxomatous changes. A significant number of fibroblasts with evidence of active proliferation were observed interspersed among the fibers, as demonstrated by immunohistochemistry with antibodies to α-SMA.

In the control group, the degree of inflammation in the thickness of the bile duct walls and adjacent connective tissue was more pronounced. This was evidenced by the presence of foci of infiltration of lymphocytes with a mixture of individual plasma cells, as confirmed by a strong expression in the reaction with antibodies to CD3. In the SVF-treated group, the degree of inflammation was less pronounced in the thickness of the duct walls and surrounding connective tissue (IHC staining for CD3). Additionally, the infiltrate in this group included cells with CD10 antigen, indicating the presence of pre-B and pre-T lymphocytes.

In the control group, the walls of the vessels were observed to have thickened, while the endothelial structure remained normal. Additionally, the density of vascularization in the operation area was noted to be low. In the SVF-treated group, the vessels exhibited a more primitive structure and a thinned endothelium. Some vessels displayed cavities lined with barely discernible endothelial cells and filled with erythrocytes. Immunohistochemical examination revealed that immature endothelial cells expressed CD34. Morphometric examination demonstrated that the vascular density in the SVF-treated group was 1.7 times higher than in the control group.

**Figure 2:**
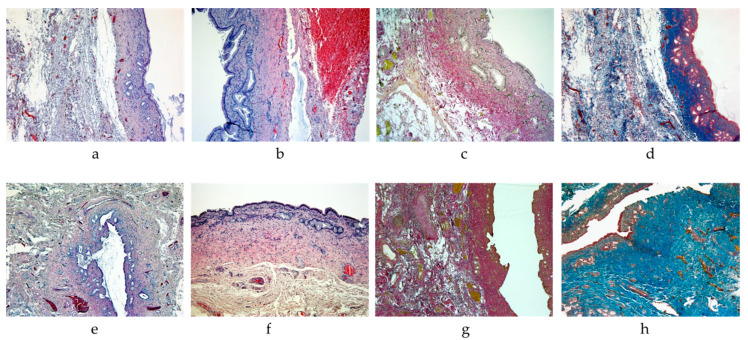
Common hepatic duct wall ((**a**–**d**)—SVF-treated group; (**e**–**h**)—control group): (**a**,**e**)—magn. ×50, Hematoxylin&Eosin staining; (**b**,**f**)—magn. ×100, Hematoxylin&Eosin staining; (**c**,**g**)—magn. ×50, Van Gieson staining; (**d**,**h**)—magn. ×50, Picro-Mallory staining. The SVF-treated group shows a decrease in fibrotic changes, decreased inflammatory cell infiltration, and improved vascularization, with connective tissue fibers showing mild fibrotic changes and increased fibroblast presence. The control group shows significant fibrotic changes with dense collagen fiber replacement, extensive hemorrhage, and moderate lymphoplasmacytic infiltration around blood vessels, along with a normal muscle layer structure with some sclerosis.

The mucosal layer of the SVF-treated group is distinguished by a simple columnar epithelium, with regions exhibiting indications of hyperplasia and the formation of multiple villi (Figure 2a). The lamina propria is loose, exhibiting an extensive proliferation of small glands with dilated ducts lined by a simple columnar epithelium. Some glands invade the muscular layer. Vessels are small, engorged, and numerous (Figure 2a,b). There is a mild infiltration of lymphocytes and isolated macrophages around the vessels, and a moderate proliferation of fibroblasts.

The muscular layer is observed to be of normal structure, with smooth myocytes exhibiting no distinctive features (see Figure 2a,b). The serous membrane displays indications of mild sclerosis. The adjacent adipose tissue in the area of the anastomosis displays partial replacement by thin, loose collagen fibers, with areas of myxomatous and hyalinosis changes in the connective tissue (stained by Van Gieson’s and by picro-Mallory’s), as showed in Figure 2c,d. A notable number of fibroblasts are observed, exhibiting indications of proliferation. The tissue displays extensive hemorrhages and dilated, engorged vessels that, in comparison to the control group, exhibit a more primitive structure and are lined with thinned endothelium. Some vessels are represented by cavities that are lined with endothelial cells that are barely discernible and filled with erythrocytes. A weakly expressed infiltration is observed around the vessels, consisting of lymphocytes, macrophages, and plasma cells (Figure 2a,b). Upon staining with picro-Mallory, the thin and loose bundles of collagen fibers in the adjacent tissue in the surgical area exhibited a light blue hue, with fibroblast nuclei displaying a reddish tint and a notable presence of fibroblasts (Figure 2d). Van Gieson staining revealed that the connective tissue fibers were bright red, indicating a mild expression of fibrotic changes (Figure 2c). Consequently, the SVF-treated group demonstrated a reduction in fibrotic alterations, a diminished infiltration of inflammatory cells, and enhanced vascularization.

The control group is distinguished by a mucosal lining comprising a simple columnar epithelium with flattened villi. The lamina propria is observed to be loose and demonstrates a mild lymphoplasmacytic infiltration, accompanied by the proliferation of small glands with dilated ducts that are lined by a simple columnar epithelium (Figure 2e). The blood vessels are sparse and engorged, surrounded by infiltration of lymphocytes and macrophages (Figure 2e,f). There is a slight increase in the number of fibroblasts. The muscle layer is of a normal structure, composed of smooth muscle cells, with some replacement by sclerosis (Figure 2f). The serous membrane displays indications of sclerosis. The adjacent adipose tissue in the area of the anastomosis has undergone significant replacement by connective tissue, comprising multidirectional bundles of dense, fibrosed, and sclerosed collagen fibers and proliferating fibroblasts within the connective tissue matrix. There are extensive hemorrhages and a small number of dilated, swollen vessels with thickened walls and normal endothelium. A perivascular focal moderate infiltration of lymphocytes, macrophages, and plasma cells is observed, with occasional fibroblasts exhibiting signs of proliferation. Picro-Mallory staining reveals the presence of multidirectional bundles of collagen fibers, which are stained dark blue in the adjacent connective tissue in the anastomotic zone (Figure 2h). Fibroblast nuclei are stained reddish, while fibrin and elastic fibers are not detected. Van Gieson’s stain demonstrates that the connective tissue fibers are bright red, indicating pronounced fibrotic changes (Figure 2g).

### 2.5. Immunohistochemical Staining Studies

**Figure 3:**
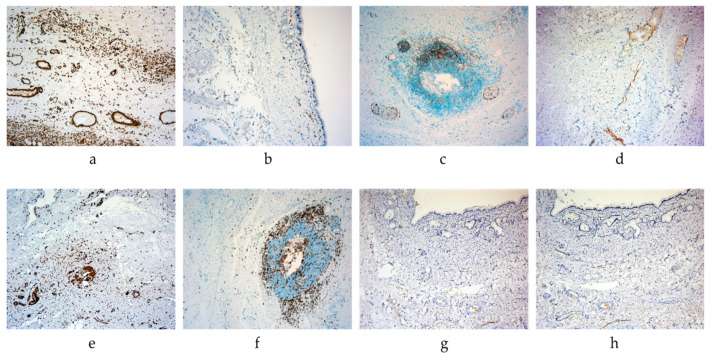
Common hepatic duct tissues, IHC study ((**a**–**d**)—SVF-treated group, (**e**–**h**)—control group): (**a**)—SVF-treated group, ×100 magn., α-SMA staining (+++); (**b**)—SVF-treated group, ×100 magn., CD3 staining (++); (**c**)—SVF-treated group, ×100 magn., CD10 staining (++); (**d**)—SVF-treated group, ×100 magn., CD34 staining (+); (**e**)—control group, ×100 magn., α-SMA staining (++); (**f**)—control group, ×100 magn., CD3 staining (+++); (**g**)—control group, bile duct wall, ×100 magnification, CD10 staining (-); (**h**)—control group, bile duct wall, ×100 magn., CD34 staining (-). The SVF-treated group was characterized by more expression in fibroblasts, less presence of CD3+ T lymphocytes, and presentation of CD10+ and CD34+ cells.

The immunohistochemical reaction with antibodies against α-SMA (alpha-smooth muscle actin) in the anastomosis zone revealed strong expression in fibroblasts (+++) in the SVF-treated group. The reaction with CD3 antibodies demonstrated moderately expressed levels in T-lymphocytes (++). Moderate expression of CD10 was observed in certain infiltrate cells (++), which can be identified as pre-B and pre-T lymphocytes. Additionally, focal weak expression of CD34 was detected in the forming endothelium of vessels (+) (see Table 2 and Figure 3).

In the control group, an immunohistochemical reaction with antibodies against α-SMA (alpha-smooth muscle actin) in the surgical intervention area demonstrated moderate expression levels in fibroblasts (++). Reaction with CD3 antibodies exhibited strong expression in T-lymphocytes within inflammatory foci (+++). No expression of CD10 and CD34 was observed (see Table 2 and Figure 3).

### 2.6. Quantitative Morphometry

**Figure 4:**
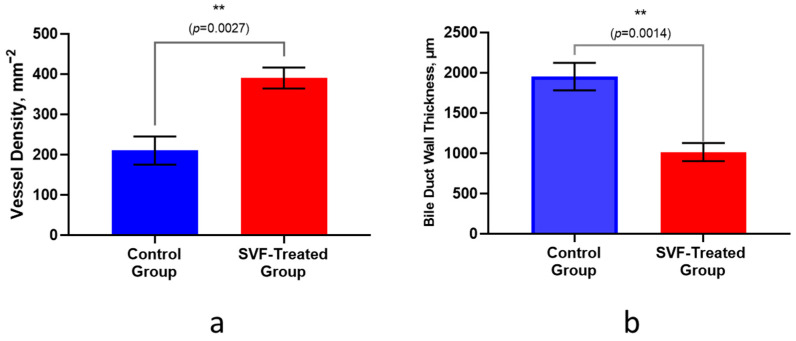
(**a**) Vessel density in the surgical area, mm^−2^, (**b**) Common Hepatic Duct Wall Thickness, µm. **—*p*-value < 0.01.

The vessel density in the surgical area was 390 vessels per 1 mm^2^ in the SVF-treated group, and 210 vessels per 1 mm^2^ in the control group (Figure 4a). The average thickness of the common hepatic duct wall was 1951.55 ± 171.17 µm in the control group, which is almost twice as much as in the SVF-treated group (1015.45 ± 113.12 µm) (Figure 4b).

### 2.7. Mast Cell Responses

**Figure 5:**
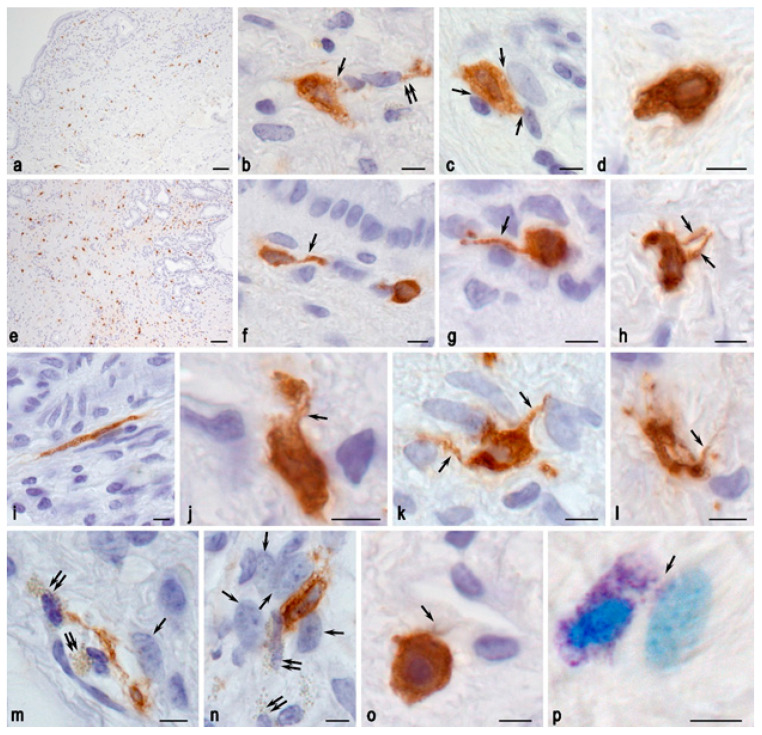
Tryptase-positive mast cells in the bile duct of pigs. Methods: (**a**–**o**)—immunohistochemical detection of tryptase, (**p**)—toluidine blue staining. Groups: (**a**–**d**) control group, (**e**–**p**) SVF-treated group. (**a**)—Distribution of mast cells of various sizes in the structural sheaths of the bile duct; (**b**)—Secretion of tryptase into the microenvironment of the bile duct stroma (indicated by an arrow), including towards a fibroblast (double arrow); (**c**)—Co-localization of a mast cell with several fibroblastic differentiation cells (indicated by an arrow), showing signs of various mechanisms of targeted tryptase secretion; (**d**)—Mast cell with high tryptase content surrounded by collagen fibers; (**e**)—High density of mast cells in the structural sheaths of the bile duct; (**f**)—Mast cell with a process (arrow) in the subepithelial layer of the lamina propria of the bile duct mucosa; (**g**)—Mast cell in the bile duct stroma with a long cytoplasmic process (arrow) contacting several cells; (**h**)—Mast cell with two cytoplasmic processes (arrow) among the collagen fibers of the stroma; (**i**)—Spindle-shaped mast cell; (**j**–**l**)—Various types of mast cell contacts with the structures of the tissue microenvironment of the bile duct through the formation of cytoplasmic extensions (arrow) and signs of secretion; (**m**,**n**)—Interaction of mast cells with eosinophils (double arrow) and fibroblasts (arrow) during extracellular matrix remodeling and angiogenesis (presumably); (**o**)—Mast cell with high tryptase content in the pericapillary tissue microenvironment showing signs of secretory activity (arrow); (**p**)—Directed secretion of small metachromatic granules from the mast cell towards a fibroblast (arrow). Scale bar: (**a**,**e**)—50 µm, others—5 µm.

The study demonstrated that the mast cells in the bile duct of pigs exhibited a distinctive morphology, characterized by a relatively small size (~10 to 12 µm) and a limited number of secretory granules. On occasion, tryptase was observed in the cytoplasm of mast cells, appearing as a nearly dust-like granularity. These morphological features evidently presented a challenge in identifying mast cells when stained with toluidine blue. In the control group, mast cells were observed throughout the entire thickness of the bile duct wall, including the mucosa (Figure 5a). They were most frequently localized with other stromal cells, including fibroblasts (Figure 5b,c). Less frequently, mast cells were found among the collagen fibers of the bile duct stroma.

The number of mast cells in the SVF-treated group was increased, but not significantly; their secretory activity was also enhanced. Mast cells were characterized by the formation of elongated cytoplasmic processes extending over considerable lengths (Figure 5f–l). Elongated mast cells were often observed (Figure 5i). Of particular note was the active interaction with eosinophils and fibroblasts (Figure 5m,n,p), as well as a more frequent localization near the vascular network (Figure 5o).

**Figure 6:**
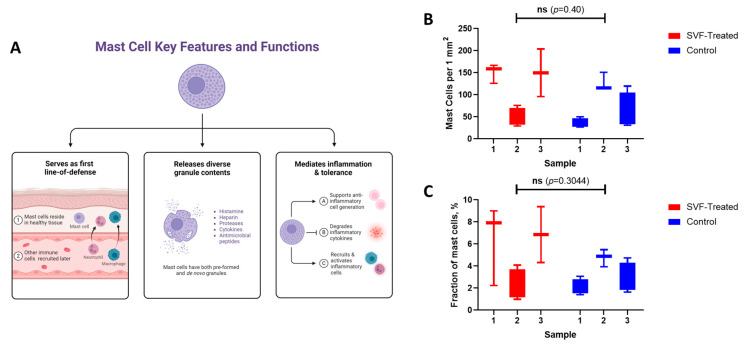
Mast cells in bile duct anastomosis healing: (**A**) Schematic of the functions of mast cells during tissue repair. Created with Biorender.com; (**B**) Presence of mast cells per 1 mm^2^; (**C**) Fraction of mast cells among all resident cells.

Mast cells play the pivotal role in bile duct repair and regeneration (Figure 6A). The injection of SVF cells in the SVF-treated group resulted in not only an increase in the population of mast cells within the organ wall (Figure 6B,C), but also in increased secretory activity and alterations in their morphological parameters. However, these changes were not found to be statistically significant when the presence of mast cells (*p* = 0.40), nor when the relative fraction of mast cells (*p* = 0.30).

## 3. Discussion

Insufficiency or stricture of the biliary anastomosis is among the most common complications following liver transplantation in clinics, occurring in the early and late postoperative periods, respectively. Bile leaks are observed in 2–25% of cases post-surgery and are categorized into two distinct periods: early (first 4 weeks) and late (beyond the 4-week period) [3,34]. The treatment of this complication is time-consuming and has a significantly detrimental impact on the patient’s quality of life. Strictures that obstruct bile flow can result in secondary liver failure, secondary biliary cirrhosis, and the necessity for retransplantation. Despite advancements in surgical techniques and instrumentation, the incidence of this complication remains high, at 5–15% in cadaveric transplantation and 4–25% in living-related donor transplantation [2,3,34]. Consequently, researchers are exploring new technologies to improve the healing conditions of anastomoses.

The potential of mesenchymal stem cells (MSCs), including bone marrow stem cells (BMSCs) and adipose tissue stem cells (ADSCs), to accelerate wound healing has been the subject of intensive investigation in recent times [35,36,37,38]. The principal functions of stem cells include the stimulation of angiogenesis, the secretion of growth factors (hepatocyte growth factor, vascular endothelial growth factor), differentiation, immunomodulatory effects, and anti-inflammatory activity [39,40]. Both BMSCs and ADSCs exhibit comparable capacities for differentiation and regeneration. However, ADSCs are more readily applicable in clinical settings due to a more straightforward and secure procurement process.

Although the acquisition of ADSCs does not present a significant technical challenge, the delivery and retention of these cells in the target area remains a complex and underdeveloped task. Several studies have reported on the local application of ADSCs, yet the long-term fate of the injected cells remains unclear [41]. The direct injection of cells into the thin wall of the bile duct represents a significant challenge, prompting some authors to propose the use of cell sheet transplantation as a potential solution. The efficacy of this technology has been demonstrated in corneal repair [42], prevention of esophageal stenosis after endoscopic submucosal dissection [43], treatment of myocardial infarction [44], prevention of pancreatic fistulas [45], and insufficiency of intestinal anastomoses [46].

The impact of mesenchymal stem cells or cell-derived sheets (MSC sheets) on the repair of bilio-biliary anastomoses in pigs has been previously investigated [31,47], and it was demonstrated that MSC sheets reduced hypertrophic changes in the bile duct wall at the anastomosis site. The histological evaluation of bilio-biliary anastomoses in the control group revealed an increase in the number of inflammatory cells and a thickening of the duct wall, which was attributed to an increase in collagen fibers.

Another notable mechanism is related to the secretory activity of SVF cells. Previously, the angiogenic effect of trophic secretion of vascular endothelial growth factors has been shown to enhance the healing process [48,49]. These newly formed vessels account for the observed differences in anastomotic healing [47,50], and in our study, the application of SVF cells increased vascular density and stimulated anastomotic healing. Follow-up immunological responses are caused by alterations in mast cell activity, which have been associated with alterations in bile duct repair and regeneration [32,33].

The results of IHC analysis provide valuable insights into the cellular dynamics and tissue remodeling processes occurring in the anastomotic zone. In the SVF-treated group, the strong expression of α-SMA in fibroblasts suggests increased myofibroblast activity, which is indicative of robust tissue remodeling and wound healing. This increased fibroblast activity could be attributed to the regenerative properties of SVF, potentially facilitating improved structural integrity and function at the anastomotic site. The moderate expression of CD3+ T lymphocytes indicates an active yet controlled immune response, which may be beneficial for tissue repair without excessive inflammation. The presence of CD10+ cells, identified as pre-B and pre-T lymphocytes, suggests a dynamic immune milieu that could contribute to tissue regeneration. In addition, the focal weak expression of CD34 in the forming endothelium suggests neovascularization, which is crucial for the supply of nutrients and oxygen to the healing tissue.

The morphofunctional rearrangements of mast cells resulted in enhanced interactions with other immunocompetent cells, including fibroblasts, fibrocytes, and myofibroblasts. It can be surmised that in this state, mast cells are able to contact a greater number of cells in the biliary tissue microenvironment simultaneously. As a result, the conditions were established for more effective synchronization of morphogenetic processes in the bile duct wall during the postoperative period. Ultimately, the superior quality and accelerated healing of the bile duct under SVF treatment conditions may be attributed to the discernible alterations in the regulatory capacity of mast cells in relation to other cellular populations and the extracellular milieu of the tissue microenvironment. It is established that mast cell tryptase possesses the capability to enhance fibroblast proliferation, stimulate their migratory potential, transform them into myofibroblasts, induce eosinophil chemotaxis, activate multiple matrix metalloproteinases, and exert angiogenic effects [51,52,53,54]. Furthermore, additional augmentation of mast cell degranulation and augmented vascular permeability are feasible [55,56]. In our study, the local application of SVF suspension in the area of the bilio-biliary anastomosis demonstrated a diminished severity of inflammatory and scarring alterations at the two-month follow-up. At this juncture, it is not feasible to ascertain whether this impact will manifest as a reduction in the incidence of strictures over the long term.

In our study, it was found that the injections of SVF into the area of bilio-biliary anastomosis did not demonstrate any adverse effects in the early postoperative period. No signs of biliary anastomosis failure were recorded in any animal in either group, which may be related to the small number of observations. SVF treatment resulted in the formation of cytoplasmic processes in mast cells, a phenomenon observed much less frequently in the control group. One of the limitations of this study was the lack of morphological clarification of the mechanisms behind the transformation of cytoplasmic processes in mast cells.

## 4. Materials and Methods

### 4.1. Experimental Animals

The study was performed on six (*n* = 6) Landrace swine (females, mass 50 ± 3 kg, 4 months old, with three animals both in the SVF-treated group, and the control group). The protocol of the study was approved by the Local ethics committee of Petrovsky National Research Centre of Surgery (protocol #5, issued 19 May 2022). The animals were in quarantine for one month prior to the study and divided into control (*n* = 3) and SVF-treated (*n* = 3) groups. Biological material for SVF separation (portion of a greater omentum) was collected in swine in the SVF-treated group under general anesthesia 24 h prior to surgery for the purpose of obtaining a cellular product. All surgical procedures were performed by the same team of surgeons.

Anesthesia was administered in accordance with humane animal care guidelines using the standard technique [57]. Zoletil-100 solution (10 mg/kg) and Xylanit-20 solution (2 mg/kg) were injected intramuscularly 30 min before surgery. Electrocardiogram electrodes and a pulse oximeter sensor were attached. During the premedication phase, a peripheral catheter was placed in the auricular vein for intravenous injections. Anesthesia was deepened through intravenous infusion of propofol solution (1.5 mg/kg). Surgery was performed under conditions of artificial lung ventilation using a Stephan Portec ventilator (F. Stephan GmbH, Gackenbach, Germany). Intubation was performed with an endotracheal tube size 6.5–7. Arduan (0.08 mg/kg) was administered intravenously as a muscle relaxant. An introducer was placed in the subclavian vein to collect blood for biochemical analysis. During surgery, propofol (5 mg/kg) and fentanyl (0.005–2.0%) were administered based on animal weight and hemodynamic parameters. Intraoperatively, heart rate, central venous pressure, and electrocardiography were monitored using the OmniCare 24C device from Hewlett-Packard (Canton, OH, USA). Intraoperative monitoring of heart rate, arterial and central venous pressures, and electrocardiography were performed. Euthanasia was medically induced in accordance with ethical standards for animal experiments and bioethical regulations [58,59].

### 4.2. Procedure for the Collection of Biological Material and the Cell Derivation

In the preparatory phase, an anesthetized animal underwent a midline mini-laparotomy, followed by the resection of a portion of the greater omentum. The collected biological material, amounting to 3 mL, was placed in a 15 mL tube containing transport medium (DMEM L/G, supplemented with penicillin at 300 U/mL and streptomycin at 300 µg/mL) and transported to the laboratory within 3 h. The isolation of the SVF was performed using classical enzymatic digestion under sterile conditions [56]. The adipose tissue was washed three times with phosphate-buffered saline (PBS) and then treated with 3 mL of 0.15% type II collagenase solution (Sigma, Livonia, MI, USA), an amount equal to the volume of the washed adipose tissue. The mixture was incubated for 30 min at 37 °C on a shaker. The obtained cell suspension was filtered through a 40 µm mesh sieve and washed three times with PBS to remove the enzymes. Quantification of viable cells in SVF suspension was performed using a Goryaev chamber by 0.1% solution of trypan blue staining (Life Technologies, Eugene, OR, USA).

### 4.3. Experimental Study Design

**Figure 7:**
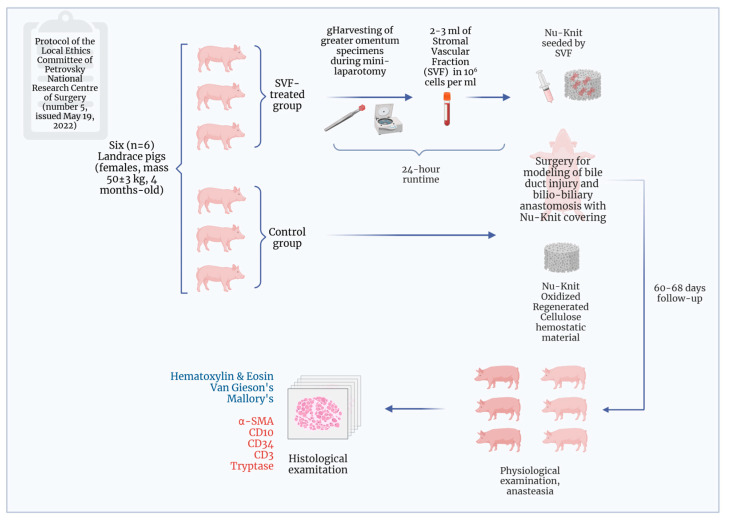
Schematic of an experimental study design. Created with Biorender.com.

The design of the experimental study is presented in Figure 7.

**Figure 8:**
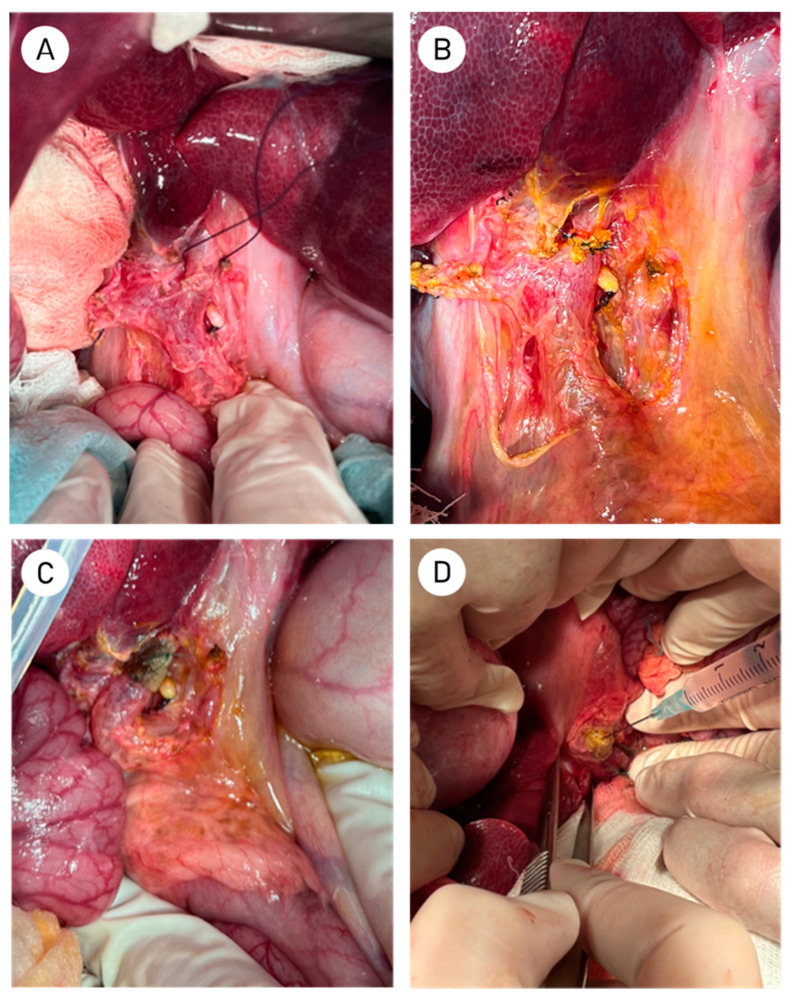
(**A**) Isolation of the common hepatic duct and securing with a traction suture; (**B**) Creation of the common hepatic duct anastomosis, Anterior wall of anastomosis is completed; (**C**) Anastomosis is wrapped with a strip of Surgicel Nu-Knit hemostatic patch; (**D**) Saturation of Surgicel Nu-Knit and periductal tissues with 2–3 mL of SVF suspension.

Following the administration of anesthesia to the animal, a midline laparotomy was conducted, and a cholecystectomy was performed. Following the cholecystectomy, the hepatoduodenal ligament was dissected, and the common hepatic duct was isolated and secured with a traction suture (Figure 8A). The common hepatic duct was then transected with a scalpel and an end-to-end anastomosis of the duct was constructed with single Maxon 6-0 sutures (Figure 8B).

Integrity of the formed anastomosis was evaluated through the application of a gauze pad [60]. In the event of bile leakage, the defect area was reinforced with additional interrupted sutures. Subsequently, the anastomosis was verified and wrapped with a Surgicel Nu-Knit Oxidized Regenerated Cellulose hemostatic material (J & J Healthcare Systems, Piscataway, NJ, USA) strip measuring approximately 15 × 20 mm [61]. The edges of the strip were then sutured together with two or three interrupted sutures, thus enhancing fixation (Figure 8C). In the SVF-treated group, 2–3 mL of SVF was injected using a syringe to saturate the Surgicel Nu-Knit and periductal tissues (Figure 8E). The subhepatic space was drained using a tubular drainage system, and the laparotomy incision was sutured. In the postoperative period, the drainage output was monitored. In the absence of bile in the drainage output, the drainage was removed.

### 4.4. Animal Anesthesia and Samples Collection

The animals were anesthetized and samples were collected 60 to 68 days after surgery. Following the administration of anesthesia to the animals, a midline laparotomy was conducted. During this procedure, the subhepatic space was visually assessed for the presence of adhesions and scar tissue, as well as for the identification of localized bile collections. The surrounding tissues were then dissected, and the common hepatic and common bile ducts were isolated for the purpose of evaluating changes in the anastomotic area. Subsequently, a segment of the common hepatic duct and common bile duct, measuring between 3 and 5 cm, was resected and prepared for morphological examination. After the tissue was collected, the animals were euthanized.

### 4.5. Morphological Assessment

The specimens were fixed in 10% neutral buffered formalin for a period of 24 h, processed in a tissue processor, and embedded in paraffin. Histological analysis was conducted on sections from the paraffin blocks, which were stained with hematoxylin and eosin, Van Gieson’s picrofuchsin, and Mallory’s phosphotungstic acid hematoxylin.

Immunohistochemical analysis was performed automatically in an immunostainer (BenchMark XT, Ventana, Roche, Germany), by antibodies for anti-α-SMA (Dako; clone 1A4; dilution 1:50), CD10 (Cell Marque, clone 56C6; dilution 1:30), CD34 (Dako; clone QBEnd-10; dilution 1:50), CD3 (Dako; clone F7.2.38, dilution 1:50), and anti-Mast Cell Tryptase antibody [AA1] (Abcam, #ab2378, dilution 1:2000). Microscopy of the specimens was carried out at magnifications of ×50, ×100, ×200, and ×400 using Leica DM5000B light microscope (Leica Microsystems, Wetzlar, Germany). Capture of histological images was performed using a Leica DFC490 camera with LAS V4.8 software (Leica Microsystems, Germany).

### 4.6. Statistical Analysis

The acquired images were analyzed using the morphometry software Axio Vision 4.8.2 (Carl Zeiss, Germany). To ensure the reliability of the data, a series of replicate measurements were employed for the determination of each parameter. The data were presented as mean values ± standard deviations (SD). The software GraphPad Prism 8 (GraphPad Software Inc., La Jolla, CA, USA) was used for statistical data analysis and for the generation of graphs. A Welch’s *t*-test was performed to assess the significance of the differences. The level of statistical significance was considered to be a *p*-value < 0.05.

## 5. Conclusions

Local injections of autologous SVF at the site of bilio-biliary anastomosis significantly enhance healing and promote tissue regeneration in the porcine model. These findings suggest that SVF application could be a valuable adjunctive therapy in bilio-biliary anastomosis surgery, potentially improving anastomosis integrity and follow-up outcomes. Further investigation is needed to explore the clinical applicability and long-term benefits of this novel approach in clinical practice as a minimally manipulated cell application.

## Figures and Tables

**Table 1:** The properties of SVF samples isolated from porcine adipose tissue of animals in the SVF-treated group.

Sample No.	#1	#2	#3
Value of adipose tissue, ml	4	3.5	3
Total count of derived cells, ×10^6^	3.6	3.85	2.94
Cell viability, %	95	92	94
Cells per 1 mL of adipose tissue, ×10^6^	0.9	1.1	0.98
Cell doubling time, hours	22	25	23

**Table 2:** Tissue morphometry in the anastomosis zone by IHC with antibodies.

IHC Marker	SVF-Treated Group	Control Group
α-SMA (α-smooth muscle actin)	Strong expression in fibroblasts (+++)	Moderate expression in fibroblasts (++)
CD3	Moderate expression in T-lymphocytes (++)	Strong expression in T-lymphocytes (+++)
CD10	Moderate expression in some cells of the infiltrate (++)	Undetected
CD34	Weak expression in vascular endothelium (+)	Undetected

## Data Availability

The raw data supporting the conclusions of this article will be made available by the authors on request.

## References

[1] Takahashi K., Nagai S., Putchakayala K.G., Safwan M., Gosho M., Li A.Y., Kane W.J., Singh P.L., Rizzari M.D., Collins K.M. (2017). Prediction of biliary anastomotic stricture after deceased donor liver transplantation: The impact of platelet counts–a retrospective study. Transpl. Int..

[2] Boeva I., Karagyozov P.I., Tishkov I. (2021). Post-liver transplant biliary complications: Current knowledge and therapeutic advances. World J. Hepatol..

[3] Jung D.H., Ikegami T., Balci D., Bhangui P. (2020). Biliary reconstruction and complications in living donor liver transplantation. Int. J. Surg..

[4] Klabukov I.D., Baranovskii D.S., Shegay P.V., Kaprin A.D. (2023). Pitfalls and promises of bile duct alternatives: There is plenty of room in the regenerative surgery. World J. Gastroenterol..

[5] Sun Q., Shen Z., Liang X., He Y., Kong D., Midgley A.C., Wang K. (2021). Progress and current limitations of materials for artificial bile duct engineering. Materials.

[6] Evstratova E., Smirnova A., Skornyakova E., Baranovskii D., Klabukov I. (2025). Recombinant collagen coating 3D printed PEGDA hydrogel tube loading with differentiable BMSCs to repair bile duct injury: The Deficiency of Engineering Approaches in Tissue Engineering Research. Colloids Surf. B Biointerfaces.

[7] Pesce A., Palmucci S., La Greca G., Puleo S. (2019). Iatrogenic bile duct injury: Impact and management challenges. Clin. Exp. Gastroenterol..

[8] Martinez-Isla A., Acosta-Mérida M.A., Navaratne L., Ashrafian H. (2022). History of Bile Duct Surgery. Laparoscopic Common Bile Duct Exploration.

[9] Jabłońska B., Lampe P. (2009). Iatrogenic bile duct injuries: Etiology, diagnosis and management. World J. Gastroenterol. WJG.

[10] Klabukov I., Tenchurin T., Shepelev A., Baranovskii D., Mamagulashvili V., Dyuzheva T., Krasilnikova O., Balyasin M., Lyundup A., Krasheninnikov M. (2023). Biomechanical Behaviors and Degradation Properties of Multilayered Polymer Scaffolds: The Phase Space Method for Bile Duct Design and Bioengineering. Biomedicines..

[11] Lee M.C., Pan C.T., Huang R.J., Ou H.Y., Yu C.Y., Shiue Y.L. (2024). Investigation of Degradation and Biocompatibility of Indirect 3D-Printed Bile Duct Stents. Bioengineering.

[12] Shestakova V.A., Klabukov I.D., Baranovskii D.S., Shegay P.V., Kaprin A.D. (2022). Assessment of immunological responses-a novel challenge in tissue engineering and regenerative medicine. Biomed. Res. Ther..

[13] Petrus-Reurer S., Romano M., Howlett S., Jones J.L., Lombardi G., Saeb-Parsy K. (2021). Immunological considerations and challenges for regenerative cellular therapies. Commun. Biol..

[14] Tekant Y., Serin K.R., İbiş A.C., Ekiz F., Baygül A., Özden İ. (2023). Surgical reconstruction of major bile duct injuries: Long-term results and risk factors for restenosis. Surgeon.

[15] Biesel E.A., Kuesters S., Chikhladze S., Ruess D.A., Hipp J., Hopt U.T., Fichtner-Feigl S., Wittel U.A. (2024). Surgical complications requiring late surgical revisions after pancreatoduodenectomy increase postoperative morbidity and mortality. Scand. J. Surg..

[16] Lan T., Qian S., Tang C., Gao J. (2022). Role of immune cells in biliary repair. Front. Immunol..

[17] Yu Y., Yue Z., Xu M., Zhang M., Shen X., Ma Z., Li J., Xie X. (2022). Macrophages play a key role in tissue repair and regeneration. PeerJ.

[18] Björkström N.K. (2022). Immunobiology of the biliary tract system. J. Hepatol..

[19] Zhao J., Yue P., Mi N., Li M., Fu W., Zhang X., Gao L., Bai M., Tian L., Jiang N. (2024). Biliary fibrosis is an important but neglected pathological feature in hepatobiliary disorders: From molecular mechanisms to clinical implications. Med. Rev..

[20] Laloze J., Fiévet L., Desmoulière A. (2021). Adipose-derived mesenchymal stromal cells in regenerative medicine: State of play, current clinical trials, and future prospects. Adv. Wound Care.

[21] Zhidu S., Ying T., Rui J., Chao Z. (2024). Translational potential of mesenchymal stem cells in regenerative therapies for human diseases: Challenges and opportunities. Stem Cell Res. Ther..

[22] Zhang J., Liu Y., Chen Y., Yuan L., Liu H., Wang J., Liu Q., Zhang Y. (2020). Adipose-Derived stem cells: Current applications and future directions in the regeneration of multiple tissues. Stem Cells Int..

[23] Semon J.A., Maness C., Zhang X., Sharkey S.A., Beuttler M.M., Shah F.S., Pandey A.C., Gimble J.M., Zhang S., Scruggs B.A. (2014). Comparison of human adult stem cells from adipose tissue and bone marrow in the treatment of experimental autoimmune encephalomyelitis. Stem Cell Res. Ther..

[24] Mazini L., Rochette L., Amine M., Malka G. (2019). Regenerative capacity of adipose derived stem cells (ADSCs), comparison with mesenchymal stem cells (MSCs). Int. J. Mol. Sci..

[25] Ryabkov M.G., Egorikhina M.N., Koloshein N.A., Petrova K.S., Volovik M.G., Orlinskaya N.Y., Moskovchenko A.O., Charykova I.N., Aleynik D.Y., Linkova D.D. (2023). Effectiveness and Safety of Transplantation of the Stromal Vascular Fraction of Autologous Adipose Tissue for Wound Healing in the Donor Site in Patients with Third-Degree Skin Burns: A Randomized Trial. Med. J. Islam. Repub. Iran.

[26] Goncharov E.N., Koval O.A., Bezuglov E.N., Engelgard M., Eremin I.I., Kotenko K.V., Ramirez M.D.J.E., Montemurro N. (2024). Comparative Analysis of Stromal Vascular Fraction and Alternative Mechanisms in Bone Fracture Stimulation to Bridge the Gap between Nature and Technological Advancement: A Systematic Review. Biomedicines.

[27] Krasilnikova O.A., Klabukov I.D., Baranovskii D.S., Shegay P.V., Kaprin A.D. (2021). The new legal framework for minimally manipulated cells expands the possibilities for cell therapy in Russia. Cytotherapy.

[28] Baranovskii D.S., Klabukov I.D., Arguchinskaya N.V., Yakimova A.O., Kisel A.A., Yatsenko E.M., Ivanov S.A., Shegay P.V., Kaprin A.D. (2022). Adverse events, side effects and complications in mesenchymal stromal cell-based therapies. Stem Cell Investig..

[29] Baranovskii D.S., Akhmedov B.G., Demchenko A.G., Krasheninnikov M.E., Balyasin M.V., Pavlova O.Y., Serova N.S., Krasil’nikova O.A., Shegai P.V., Kaprin A.D. (2022). Minimally Manipulated Bone Marrow-Derived Cells Can Be Used for Tissue Engineering In Situ and Simultaneous Formation of Personalized Tissue Models. Bull. Exp. Biol. Med..

[30] Prins H.J., Schulten E.A., Ten Bruggenkate C.M., Klein-Nulend J., Helder M.N. (2016). Bone regeneration using the freshly isolated autologous stromal vascular fraction of adipose tissue in combination with calcium phosphate ceramics. Stem Cells Transl. Med..

[31] Hara T., Soyama A., Adachi T., Kobayashi S., Sakai Y., Maruya Y., Kugiyama T., Hidaka M., Okada S., Hamada T. (2020). Ameliorated healing of biliary anastomosis by autologous adipose-derived stem cell sheets. Regen. Ther..

[32] Francis H., Meininger C.J. (2010). A review of mast cells and liver disease: What have we learned?. Dig. Liver Dis..

[33] Trussoni C.E., O’Hara S.P., LaRusso N.F. (2022). Cellular senescence in the cholangiopathies: A driver of immunopathology and a novel therapeutic target. Semin. Immunopathol..

[34] Chiche L., Guieu M., Bachellier P., Suc B., Soubrane O., Boudjema K., Navarro F., Adam R., Vaillant J.-C., Salame E. (2022). Liver transplantation for iatrogenic bile duct injury during cholecystectomy: A French retrospective multicenter study. HPB.

[35] Xie Z., Yu W., Ye G., Li J., Zheng G., Liu W., Lin J., Su Z., Che Y., Ye F. (2022). Single-cell RNA sequencing analysis of human bone-marrow-derived mesenchymal stem cells and functional subpopulation identification. Exp. Mol. Med..

[36] Ma D.H.-K., Hsueh Y.-J., Ma K.S.-K., Tsai Y.-J., Huang S.-F., Chen H.-C., Sun C.-C., Kuo M.-T., Chao A.-S., Lai J.-Y. (2021). Long-term survival of cultivated oral mucosal epithelial cells in human cornea: Generating cell sheets using an animal product-free culture protocol. Stem Cell Res. Ther..

[37] Ohki T., Ota M., Takagi R., Okano T., Yamamoto M. (2023). Long-term outcomes of regenerative treatment by endoscopic oral mucosal epithelial cell sheet transplantation for the prevention of esophageal stricture after endoscopic resection. J. Immunol. Regen. Med..

[38] Iwamoto K., Saito T., Takemoto Y., Ueno K., Yanagihara M., Furuya-Kondo T., Kurazumi H., Tanaka Y., Taura Y., Harada E. (2021). Autologous transplantation of multilayered fibroblast sheets prevents postoperative pancreatic fistula by regulating fibrosis and angiogenesis. Am. J. Transl. Res..

[39] Guillamat-Prats R. (2021). The role of MSC in wound healing, scarring and regeneration. Cells.

[40] Zhao K., Lin R., Fan Z., Chen X., Wang Y., Huang F., Xu N., Zhang X., Xuan L., Wang S. (2022). Mesenchymal stromal cells plus basiliximab, calcineurin inhibitor as treatment of steroid-resistant acute graft-versus-host disease: A multicenter, randomized, phase 3, open-label trial. J. Hematol. Oncol..

[41] Joo H.H., Jo H.J., Jung T.D., Ahn M.S., Bae K.B., Hong K.H., Kim J., Kim J.T., Kim S.H., Yang Y.I. (2012). Adipose-derived stem cells on the healing of ischemic colitis: A therapeutic effect by angiogenesis. Int. J. Color. Dis..

[42] Shang Q., Chu Y., Li Y., Han Y., Yu D., Liu R., Zheng Z., Song L., Fang J., Li X. (2020). Adipose-derived mesenchymal stromal cells promote corneal wound healing by accelerating the clearance of neutrophils in cornea. Cell Death Dis..

[43] Li M., Yang T., Zhao J., Ma X., Cao Y., Hu X., Zhao S., Zhou L. (2024). Cell sheet formation enhances the therapeutic effects of adipose-derived stromal vascular fraction on urethral stricture. Mater. Today Bio.

[44] Liu Y., Wang M., Yu Y., Li C., Zhang C. (2023). Advances in the study of exosomes derived from mesenchymal stem cells and cardiac cells for the treatment of myocardial infarction. Cell Commun. Signal..

[45] Kostecka A., Kalamon N., Skoniecka A., Koczkowska M., Skowron P.M., Piotrowski A., Pikuła M. (2024). Adipose-derived mesenchymal stromal cells in clinical trials: Insights from single-cell studies. Life Sci..

[46] Maruya Y., Kanai N., Kobayashi S., Koshino K., Okano T., Eguchi S., Yamato M. (2017). Autologous adipose-derived stem cell sheets enhance the strength of intestinal anastomosis. Regen. Ther..

[47] Hosseiniasl S.M., Felgendreff P., Tharwat M., Amiot B., AbuRmilah A., Minshew A.M., Bornschlegl A.M., Jalan-Sakrikar N., Smart M., Dietz A.B. (2023). Biodegradable biliary stents coated with mesenchymal stromal cells in a porcine choledochojejunostomy model. Cytotherapy.

[48] Kallmeyer K., André-Lévigne D., Baquié M., Krause K.H., Pepper M.S., Pittet-Cuénod B., Modarressi A. (2020). Fate of systemically and locally administered adipose-derived mesenchymal stromal cells and their effect on wound healing. Stem Cells Transl. Med..

[49] Kulakov A., Kogan E., Brailovskaya T., Vedyaeva A., Zharkov N., Krasilnikova O., Krasheninnikov M., Baranovskii D., Rasulov T., Klabukov I. (2021). Mesenchymal stromal cells enhance vascularization and epithelialization within 7 days after gingival augmentation with collagen matrices in rabbits. Dent. J..

[50] Zhang Y., Sharma A., Joo D.J., Nelson E., AbuRmilah A., Amiot B.P., Boyer C.J., Alexander J.S., Jalan-Sakrikar N., Martin J. (2020). Autologous adipose tissue–derived mesenchymal stem cells introduced by biliary stents or local immersion in porcine bile duct anastomoses. Liver Transplant..

[51] Atiakshin D., Buchwalow I., Samoilova V., Tiemann M. (2018). Tryptase as a polyfunctional component of mast cells. Histochem. Cell Biol..

[52] Elieh Ali Komi D., Kuebler W.M. (2022). Significance of Mast Cell Formed Extracellular Traps in Microbial Defense. Clin. Rev. Allergy Immunol..

[53] Ma C., Li H., Lu S., Li X., Wang S., Wang W. (2023). Tryptase and Exogenous Trypsin: Mechanisms and Ophthalmic Applications. J. Inflamm. Res..

[54] Caughey G.H. (2023). Update on Mast Cell Proteases as Drug Targets. Immunol. Allergy Clin. N. Am..

[55] O’Connell M.P., Lyons J.J. (2022). Resolving the genetics of human tryptases: Implications for health, disease, and clinical use as a biomarker. Curr. Opin. Allergy Clin. Immunol..

[56] Ong W.K., Chakraborty S., Sugii S. (2021). Adipose tissue: Understanding the heterogeneity of stem cells for regenerative medicine. Biomolecules.

[57] Zoletil Dosage Guideline. https://www.vetpharm.uzh.ch/TAK/PDFSPC/06000000/06240501-LF-EN.pdf.

[58] Ausems E.J. (1986). The European Convention for the Protection of Vertebrate Animals Used for Experimental and Other Scientific Purposes. Z. Fur. Vers..

[59] Forbes D., Blom H., Kostomitsopoulos N., Moore G., Perretta G. (2007). FELASA Euroguide: On the Accommodation and Care of Animals Used for Experimental and Other Scientific Purposes.

[60] Wang L., Yang B., Jiang H., Wei L., Zhao Y., Chen Z., Chen D. (2023). Individualized Biliary Reconstruction Techniques in Liver Transplantation: Five Years’ Experience of a Single Institution. J. Gastrointest. Surg..

[61] Simo K.A., Hanna E.M., Imagawa D.K., Iannitti D.A. (2012). Hemostatic agents in hepatobiliary and pancreas surgery: A review of the literature and critical evaluation of a novel carrier-bound fibrin sealant (TachoSil). Int. Sch. Res. Not..

